# A First-Principles Investigation of the Structural, Electronic, Optical, and Mechanical Properties of Hydrogen Storage Ordered Vacancy Double Perovskite X_2_MH_6_ Materials

**DOI:** 10.3390/nano15171339

**Published:** 2025-09-01

**Authors:** Jing Luo, Qun Wei, Xinyu Wang, Meiguang Zhang, Bing Wei

**Affiliations:** 1 School of Physics, Xidian University, Xi’an 710071, China; 2 College of Physics and Optoelectronic Technology, Baoji University of Arts and Sciences, Baoji 721016, China

**Keywords:** perovskites, hydrogen storage, optical properties, mechanical properties, first-principles calculation

## Abstract

The rising demand for clean energy, especially hydrogen, has heightened the need for efficient storage materials. Perovskites, with their unique structures, show great promise for hydrogen storage and optical uses. To identify promising candidates for hydrogen storage materials, the mechanical, electronic, and optical properties of four ordered vacancy double perovskite structures X_2_MH_6_ (Ba_2_BeH_6_, Ba_2_MgH_6_, Ca_2_BeH_6_, and Sr_2_MgH_6_) were predicted using density functional theory. These materials were confirmed to be stable, and their hydrogen storage capacity, mechanical properties, electronic structures, and optical performance were thoroughly analyzed. Ca_2_BeH_6_ demonstrated the highest gravimetric (6.32%) and volumetric (32.29 g·H_2_/L) hydrogen storage capacity, showcasing its exceptional potential. It should be noted that the hydrogen storage capacities reported here are theoretical estimates based solely on structural models, and this study does not assess the practical storage and delivery performance of these materials. Its mechanical stiffness and near-isotropic properties further enhance its practicality. Electrical studies revealed all four materials are semiconductors, all of them are direct semiconductors. Optical properties were analyzed via dielectric functions, offering key insights for designing advanced hydrogen storage and optical materials.

## 1. Introduction

The extensive use of traditional fossil fuels has caused substantial environmental damage, particularly through carbon dioxide emissions from these fuels, which have significantly contributed to the global warming crisis [1,2]. The growing global demand for clean energy has accelerated the development of hydrogen as a promising energy carrier. Despite its remarkable storage capacity under ambient conditions [3], the efficient storage of hydrogen in solid-state materials remains challenging due to its inherently low density and flammability [4]. To address these challenges, research has increasingly focused on safer and more practical solid-state hydrogen storage methods, including metals, their complexes, and metal-organic frameworks [5,6]. In recent years, the scientific community has extensively studied solid-state hydrogen storage materials, including metal hydrides and complex hydrides, in the quest for materials with higher hydrogen storage efficiency and capacity [7,8,9]. Among these, perovskite materials, particularly ordered vacancy double perovskite composites, have attracted considerable attention for their outstanding hydrogen storage properties, including high capacity, excellent reversibility, stability, and unique structural and chemical versatility [10,11,12,13,14,15]. Ordered vacancy double perovskite structures, including X_2_MH_6_, which are considered strong contenders for sustainable energy solutions due to their unique structural features and versatile applications. For example, X_2_FeH_6_ (X = Ca and Sr) [3] demonstrates excellent hydrogen storage capacity, along with favorable mechanical and optical properties, making it a highly promising hydrogen storage material. Similarly, Be_2_XH_6_ (X = Cr and Mn) [16] has attracted significant attention for its remarkable gravimetric hydrogen density, reaching up to 7.9 wt%.

Perovskite structures exhibit exceptional physical properties, encompassing remarkable structural, optical, electrical, and magnetic characteristics, as well as adjustable band gaps. These attributes provide them with a high light absorption capacity, rendering them extremely versatile for diverse applications, surpassing the capabilities of other materials [17]. Notably, ordered vacancy double perovskite structures, such as Cs_2_PtI_6_ and Rb_2_PtI_6_, have been recognized for their extraordinary optical properties [18]. Moreover, LiMgH_3_ is considered a highly promising hydrogen storage optoelectronic material due to its excellent static refractive index, dielectric properties, and outstanding gravimetric and volumetric hydrogen storage capacities [19]. To identify hydrogen storage materials with superior properties, this study systematically investigates potential candidates with ordered vacancy double perovskite structures, namely X_2_MH_6_ (X, M = Be, Mg, Ca, Sr, and Ba). Using DFT calculations, we excluded structures with positive formation energies, those that did not satisfy the Born stability criteria, or those exhibiting imaginary phonon frequencies. As a result, five stable compounds were identified: Ba_2_BeH_6_, Ba_2_CaH_6_, Ba_2_MgH_6_, Ca_2_BeH_6_, and Sr_2_MgH_6_. We analyzed their structural, electronic, optical, mechanical, and hydrogen storage properties. The findings of this study aim to provide valuable guidance for the rational design and optimization of novel metal hydrides. These advancements are intended to enhance hydrogen storage efficiency, thereby making significant contributions to the development of sustainable energy technologies.

## 2. Computational Methods

We used the Vienna Ab initio Simulation Package (VASP) to perform relaxation and simulate the relevant properties of the five structures under investigation [20]. The projector-augmented wave (PAW) potential [21], in conjunction with the generalized gradient approximation and the Perdew–Burke–Ernzerhof (PBE) functional for exchange and correlation [22], the cutoff energy for the plane-wave expansion is set to 400 eV, and a Monkhorst–Pack *k*-grid [23] with 8 × 8 × 8 points was utilized to achieve adequate convergence of the total energy, constrained to 1 × 10^−5^ eV per atom. The single-crystal elastic constants were calculated from the strain–stress relationship derived by subjecting the material to six distinct finite strains [24]. Dynamic stability was evaluated using the finite displacement technique, and the phonon spectrum was calculated using the PHONOPY package [25].

## 3. Results and Discussion

**Figure 1:**
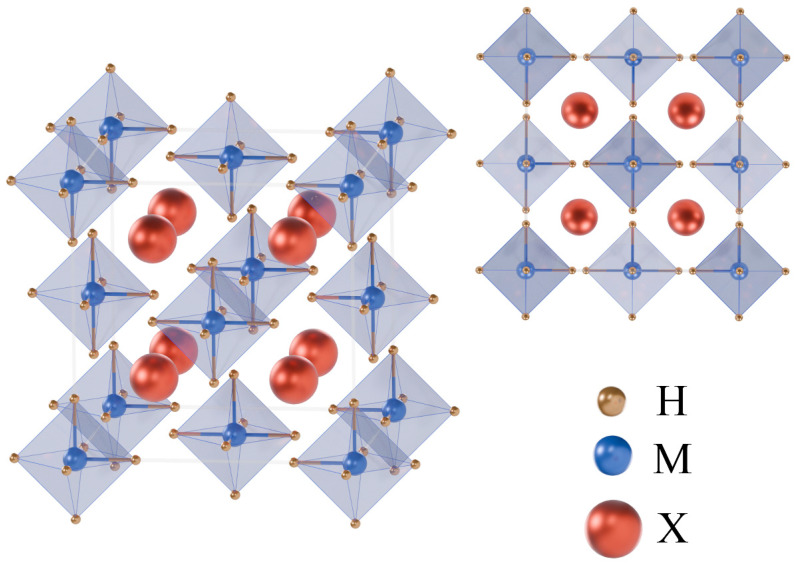
Crystal structures of the X_2_MH_6_.

Ordered vacancy double perovskite structures of X_2_MH_6_ (Ba_2_BeH_6_, Ba_2_CaH_6_, Ba_2_MgH_6_, Ca_2_BeH_6_, and Sr_2_MgH_6_) exhibit a distinctive face-centered cubic arrangement. This arrangement consists of octahedral MH_6_ units, where M represents a metal atom. The calculated M–H bond lengths in these five structures are 1.64 Å (Be–H in Ba_2_BeH_6_), 2.13 Å (Ca–H in Ba_2_CaH_6_), 1.92 Å (Mg–H in Ba_2_MgH_6_), 1.60 Å (Be–H in Ca_2_BeH_6_), and 1.90 Å (Mg–H in Sr_2_MgH_6_), respectively. The X atoms form a cubic skeleton in the perovskite lattice. The MH_6_ octahedral unit is a defining characteristic of these materials, with M denoting a metal atom. Detailed structural parameters are shown in Figure 1 and Table 1. Before evaluating their physical properties, a comprehensive stability assessment of these five structures is essential. This assessment encompasses three key criteria: thermodynamic stability, mechanical stability, and dynamical stability.

The thermodynamic stability of these structures was assessed by calculating their formation energies using the formation energy formula [26]. The formation energy ΔH is expressed as:

(1) $${\Delta H}_{f}=\left[E\left(X_{2}MH_{6}\right)-2E\left(X\right)-E\left(M\right)-6E\left(H\right)\right]/9$$

Here, ΔH  represents the formation energy of the material, while *E*(X_2_MH_6_) refers to the total energy of the structure. *E*(X), *E*(M), and *E*(H) represent the average energies of X, M, and H atoms, respectively. The results presented in Table 1, show negative formation energies for all five structures, confirming their thermodynamic stability.

**Figure 2:**
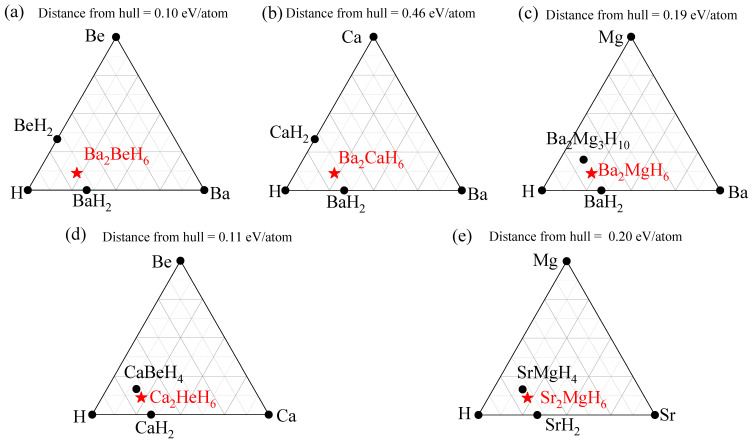
Ternary convex hull of the (**a**) Ba_2_BeH_6_, (**b**) Ba_2_CaH_6_, (**c**) Ba_2_MgH_6_, (**d**) Ca_2_BeH_6_, and (**e**) Sr_2_MgH_6_.

In addition to calculating the formation energies, we further evaluated the thermodynamic stability of these structures by computing their energy above the ternary convex hull using data from the Open Quantum Materials Database (OQMD) [27,28], as shown in Figure 2. The calculated energy above hull values for Ba_2_BeH_6_, Ba_2_CaH_6_, Ba_2_MgH_6_, Ca_2_BeH_6_, and Sr_2_MgH_6_ are 0.10, 0.46, 0.19, 0.11, and 0.20 eV/atom, respectively. These results indicate that all five compounds lie above the convex hull and are therefore thermodynamically metastable, which is commonly observed in hydrogen-rich systems. Among them, Ba_2_CaH_6_ exhibits the highest energy above hull (0.46 eV/atom), suggesting that it is less likely to be synthesized under equilibrium conditions unless specific kinetic pathways or non-equilibrium synthesis techniques are employed.

The Born criterion was used to evaluate the mechanical stability of the structure. For cubic crystals, this criterion is expressed as follows:

(2) $$C_{11}-C_{12}>0,\;C_{11}+2C_{12}>0,C_{44}>0$$

**Figure 3:**
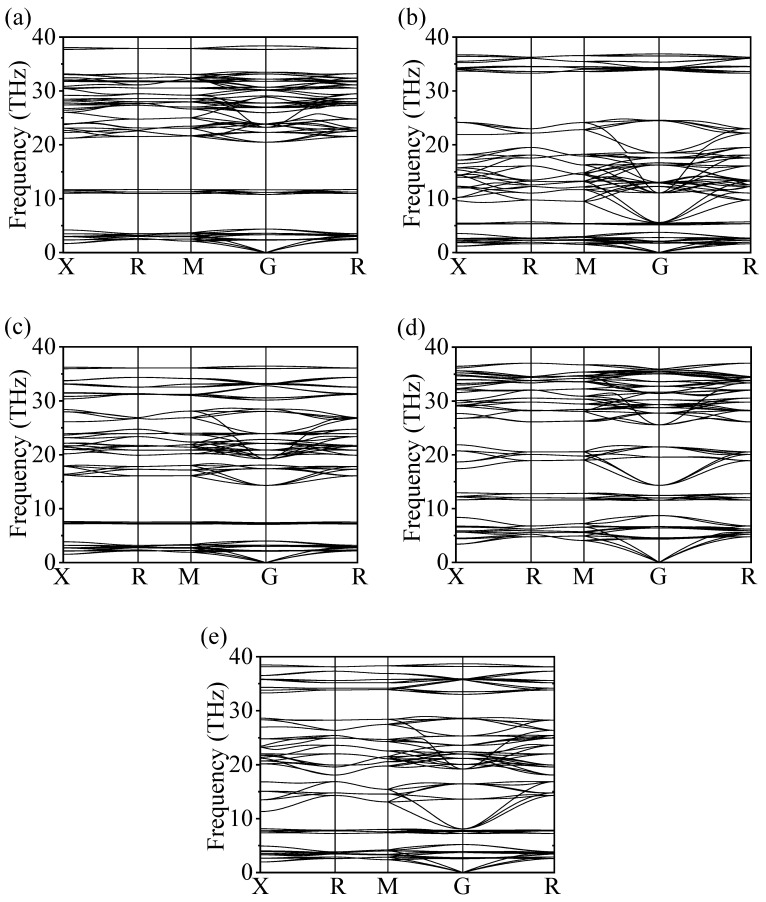
Phonon spectra of the (**a**) Ba_2_BeH_6_, (**b**) Ba_2_CaH_6_, (**c**) Ba_2_MgH_6_, (**d**) Ca_2_BeH_6_, and (**e**) Sr_2_MgH_6_.

The calculated elastic constants *C*_ij_ in Table 1 demonstrate that each of the five structures satisfy the Born criterion, thereby verifying their mechanical stability. Dynamic stability was verified by analyzing the phonon spectra of these structures shown in Figure 3 to assess their dynamic stability. No imaginary frequencies were observed below zero, confirming that all structures are dynamically stable.

**Figure 4:**
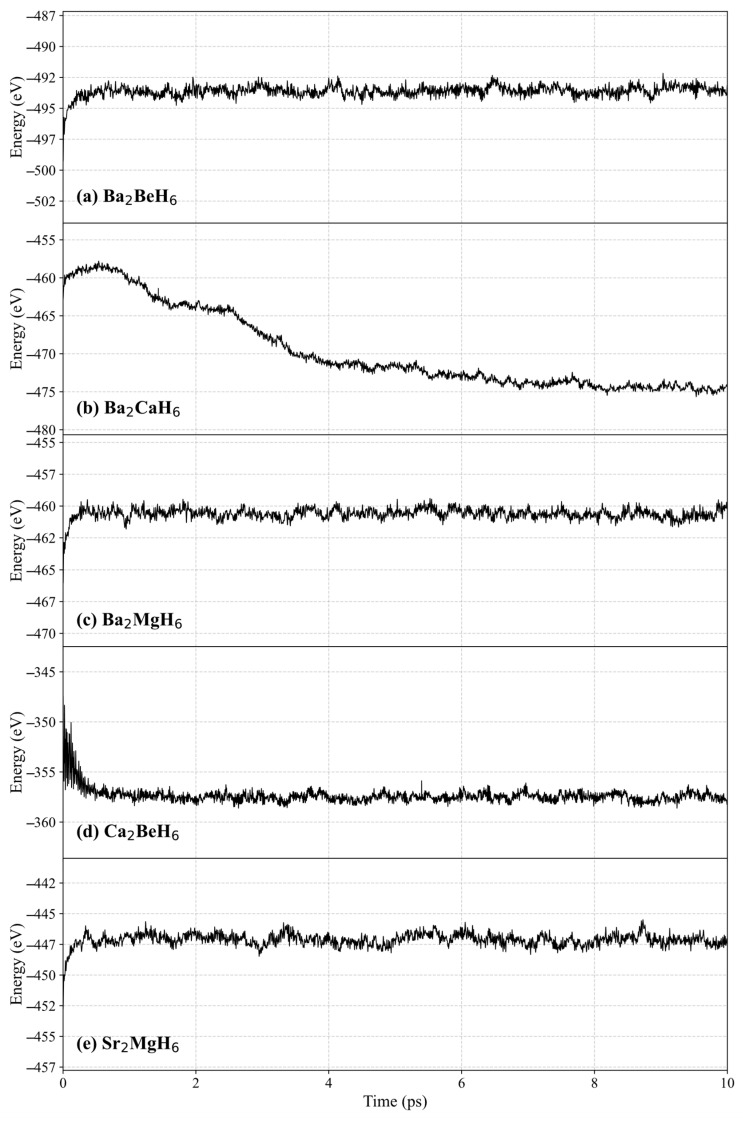
AIMD at 300 K for the (**a**) Ba_2_BeH_6_, (**b**) Ba_2_CaH_6_, (**c**) Ba_2_MgH_6_, (**d**) Ca_2_BeH_6_, and (**e**) Sr_2_MgH_6_.

Thermal stability at room temperature is critically important for energy materials intended for practical applications. Therefore, ab initio molecular dynamics (AIMD) simulations are essential for assessing their thermodynamic behavior. We performed AIMD simulations at 300 K for a duration of 10 ps, and the results are presented in Figure 4. The total energies of Ba_2_BeH_6_, Ba_2_MgH_6_, Ca_2_BeH_6_, and Sr_2_MgH_6_ remained relatively stable after approximately 2 ps, indicating good thermal stability at room temperature. In contrast, the energy of Ba_2_CaH_6_ continuously decreased throughout the simulation, suggesting a structural transformation and implying that this phase is thermodynamically unstable at 300 K.

After establishing the stability of these structures, the hydrogen storage potential of the five structures was assessed. The gravimetric hydrogen storage potential plays a crucial role in assessing the efficiency and capacity of hydrogen storage [29]. Gravimetric hydrogen storage capacity ($C_{wt}\%$) is one of the criteria for evaluating the hydrogen storage ability of materials [30]. It refers to the quantity of hydrogen stored per unit mass of the material, as expressed by the following formula [31]:

(3) $$C_{wt}\%\;=\;\left[\frac{nM_{H}}{nM_{H}+M_{Host}}\right]\times 100\%$$

Here, *n* denotes the ratio of hydrogen atoms to host compound atoms (*H*/*M*), where *M*_H_ denotes the molar mass of hydrogen and *M*_Host_ indicates the host compound’s molar mass. The calculated results are presented in Table 2, where the $C_{wt}\%$ of Ca_2_BeH_6_ reaches a remarkable 6.32%, surpassing the 4.5% target established by the U.S. Department of Energy (DOE) for rechargeable devices [32], highlighting its high potential for practical application.

The volumetric storage capacity quantifies the amount of hydrogen stored in a given volume and serves as a crucial metric for assessing a material’s hydrogen storage capability. Its mathematical expression is as follows [33]:

(4) $$\rho_{vol}\;=\;\frac{N_{H}\cdot m_{H}}{V\left(L\right)\cdot N_{A}}$$

where *N_H_* denotes the number of absorbed hydrogen atoms, *m_H_* represents the molecular mass of hydrogen, $V\left(L\right)$ corresponds to the volume of the absorbent, and *N_A_* is Avogadro’s number. The volumetric hydrogen storage capacities, $\rho_{vol}$ (in g·H_2_/L), are listed in Table 2. Among these five structures, Ca_2_BeH_6_ exhibits the highest hydrogen storage capacity due to its smaller volume.

Predicting the mechanical properties of these materials is essential for their practical application as hydrogen storage materials. Elastic parameters are critical in analyzing the mechanical behavior of the X_2_MH_6_ structure. From these constants, specific mechanical properties of the structure, including elastic anisotropy *A*, bulk modulus *B*, Young’s modulus *E*, shear modulus *G*, and Poisson’s ratio ν, can be derived from the following equations [34]:

(5) $$A\;=\;\frac{2C_{44}}{C_{11}-C_{12}}$$

(6) $$B=\frac{C_{11}+2C_{12}}{3}$$

(7) $$G_{V}=\frac{C_{11}-C_{12}+3C_{44}}{5}$$

(8) $$G_{R}=\frac{5C_{44}(C_{11}-C_{12})}{4C_{44}+3(C_{11}-C_{12})}$$

(9) $$G=\frac{G_{V}+G_{R}}{2}$$

(10) $$E=\frac{9BG}{3B+G}$$

(11) $$v=\frac{3B-2G}{6B+2G}$$

The predicted elastic moduli and Poisson’s ratios for all X_2_MH_6_ structures are shown in Table 3. Our calculations indicate that Ca_2_BeH_6_ demonstrates exceptional performance regarding the bulk modulus (67.9 GPa), shear modulus (62.3 GPa), and Young’s modulus (143.1 GPa), demonstrating exceptional mechanical stability. The higher bulk modulus suggests that Ca_2_BeH_6_ may exhibit greater stability under high-pressure conditions, while the larger shear modulus and Young’s modulus indicate that Ca_2_BeH_6_ can maintain strong rigidity under tensile or shear stress. These properties make Ca_2_BeH_6_ a potential material for hydrogen storage applications. The Poisson’s ratios of the five structures are relatively low, with Ca_2_BeH_6_ structure having the lowest Poisson’s ratio (0.149), suggesting that it experiences minimal lateral strain under stress and exhibits strong rigidity.

As shown in Table 3, compared with the two known types of hydrogen storage materials, XAlH_3_ and XScH_3_ (X = K, Rb, Cs), the X_2_MH_6_ series exhibits significantly superior mechanical properties in terms of elastic constants. Firstly, the bulk modulus (*B*) of X_2_MH_6_ materials generally falls in the range of 37.4–67.9 GPa, which is considerably higher than that of the XAlH_3_ compounds (with a maximum of only 7.05 GPa) and the XScH_3_ series (with a maximum of 40.41 GPa), indicating a stronger resistance to volume compression. Secondly, the shear modulus (*G*) and Young’s modulus (*E*) of X_2_MH_6_ are also much greater than those of the reference materials. For instance, Ca_2_BeH_6_ exhibits a *G* of 62.3 GPa and an *E* of 143.1 GPa, both substantially exceeding the maximum values of XScH_3_ (*G* = 29.41 GPa, *E* = 70.72 GPa), suggesting enhanced shear resistance and stiffness. Moreover, the *B*/*G* ratios of X_2_MH_6_ mostly lie in the range of 1.1–1.4, near the critical boundary between ductility and brittleness, while the relatively low Poisson’s ratios (ν) imply that these compounds possess both decent toughness and high hardness. Overall, the X_2_MH_6_ materials demonstrate outstanding mechanical strength and structural stability, highlighting their potential as high-performance hydrogen storage candidates.

The *B/G* ratio serves as an indicator of a material’s resistance to fracture or deformation, offering valuable insight into its bonding nature [34]. Materials with a *B/G* ratio lower than 1.75 are generally considered brittle, whereas those with a ratio exceeding 1.75 are typically regarded as ductile, suggesting the material can withstand substantial plastic deformation before fracture [37,38]. According to Table 3, the *B*/*G* ratios for the five materials range from 1.1 to 1.4. Although these values suggest limited ductility according to Pugh’s criterion, the materials exhibit sufficient mechanical strength and stability, making them suitable for hydrogen storage applications that require structural robustness.

Vickers hardness was calculated using Chen’s hardness model [39]: $H_{Chen}={2(k^{2}G)}^{0.585}-3$, where k =G/B. The results obtained from the calculations are presented in Table 3. Among these five structures, Ca_2_BeH_6_ exhibits the highest hardness at 17.1 GPa, while Ba_2_MgH_6_ shows the lowest at 8.5 GPa. The hardness variation trends of all materials are consistent with those of the shear modulus and Pugh’s modulus ratio, both of which are considered key indicators for evaluating high material hardness [40]. Additionally, Table 3 presents the melting points (*T_m_*) of these perovskite structures, and the melting points of these structures were calculated using the following formula based on the elastic constants [41].

(12) $$T_{m}=[553+5.911\times C_{12}]\pm 300$$

**Figure 5:**
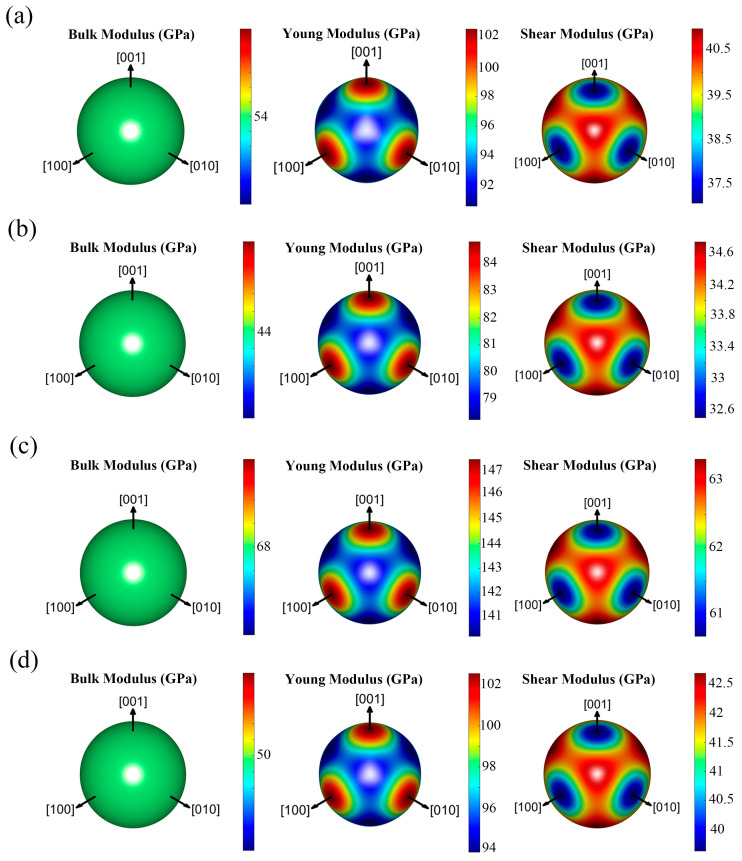
B-anisotropy, E-anisotropy and G-anisotropy diagrams of the (**a**) Ba_2_BeH_6_, (**b**) Ba_2_MgH_6_, (**c**) Ca_2_BeH_6_, and (**d**) Sr_2_MgH_6_.

The elastic anisotropy coefficient *A* quantifies the extent of anisotropy within a material. When *A* = 1, the material is perfectly isotropic. Table 3 indicates that all five materials demonstrate weak anisotropy, with Ca_2_BeH_6_ being nearly isotropic owing to its *A* value being close to 1. To better illustrate the anisotropy of these structures, anisotropy diagrams for their bulk modulus, Young’s modulus and shear modulus are depicted in Figure 5. Typically, spherical three-dimensional *B*, *E* and *G* anisotropy diagrams are used to represent ideal isotropic materials, with the distance from the surface to the center indicating the corresponding magnitudes of *B*, *E* and *G* at that location. The bulk modulus of all structures is isotropic, which is consistent with the fact that these five structures are characterized by high anisotropy coefficients *A*. The shear modulus of all five structures is anisotropic, with a similar degree of anisotropy observed across the structures. In all cases, the shear modulus along the three coordinate axes is significantly lower than in other directions. The differences in anisotropy coefficients are primarily reflected in the Young’s modulus. The Young’s modulus diagram of Ca_2_BeH_6_ is closest to a spherical shape, while that of Ba_2_BeH_6_ exhibits pronounced protrusions along the three coordinate axes, indicating significantly higher Young’s modulus values in these directions.

In summary, we systematically studied four thermodynamically stable X_2_MH_6_ structures (Ba_2_BeH_6_, Ba_2_MgH_6_, Ca_2_BeH_6_, and Sr_2_MgH_6_), focusing on their stability, mechanical properties, and hydrogen storage performance. Ca_2_BeH_6_ stands out with the lowest formation energy, excellent mechanical strength, and the highest gravimetric hydrogen storage capacity, surpassing practical targets. Its low Poisson’s ratio and high hardness indicate good durability under pressure and cycling. While the other three show slightly lower performance, they still possess promising storage capacity and stability. This work offers valuable guidance for designing efficient, eco-friendly metal hydride hydrogen storage materials.

**Figure 6:**
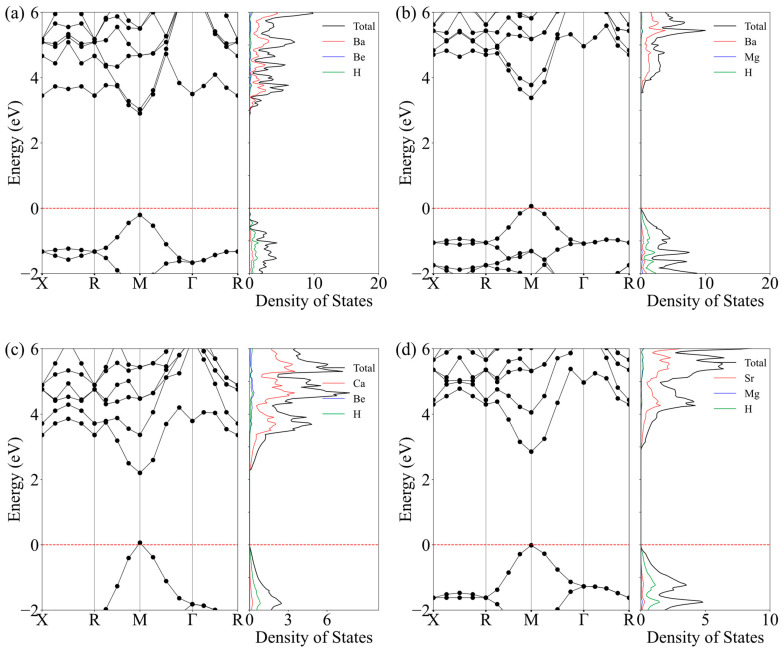
Electronic band structures and the densities of stats (States/eV) of the (**a**) Ba_2_BeH_6_, (**b**) Ba_2_MgH_6_, (**c**) Ca_2_BeH_6_, and (**d**) Sr_2_MgH_6_. The red dashed line represents the Fermi level.

Due to their tunable bandgap, perovskite materials are highly versatile and applicable in various processes, such as photovoltaic and photocatalytic applications [42]. Therefore, the band structures of these five structures have been plotted to analyze their electronic properties, as illustrated in Figure 6. The valence and conduction bands are clearly separated across all five structures, with calculated band gaps of 3.11 eV for Ba_2_BeH_6_, 3.32 eV for Ba_2_MgH_6_, 2.14 eV for Ca_2_BeH_6_, and 2.87 eV for Sr_2_MgH_6_. These values indicate that the materials exhibit semiconductor characteristics. The Conduction Band Minimum and Valence Band Maximum of the four structures are positioned at the same Γ point, demonstrating direct band gap characteristics.

The density of states (DOS) plots for these five structures were plotted to assess the influence of each atom on the electronic bands. In the valence band, the hydrogen atoms’ s-orbitals are predominant, emphasizing their critical role in determining the distribution of valence electrons in X_2_MH_6_. In contrast, the electronic states of the X atoms’ s-orbitals make a significant contribution in the valence band, suggesting that the conduction band is primarily attributed to the high-energy orbitals of the X atoms. In comparison, the contribution from the M atoms is relatively minor, primarily located at the higher energy levels of the conduction band.

Perovskites possess excellent optical properties, and certain optical parameters such as the absorption coefficient, reflectivity, extinction coefficient, energy-loss spectrum, and refractive index are closely related to the electronic band structure of the material. These properties can help reveal electronic transitions and energy level distributions within the material. Moreover, for hydrogen storage materials, the characteristics of the electronic structure influence hydrogen adsorption and release processes. Therefore, studying the optical properties provides a valuable complement for a deeper understanding of hydrogen storage performance. Therefore, the optical characteristics of these five structures will be examined next. The dielectric function (DF), which governs the optical properties of the structure, can be computed using the following equation [43]:

(13) $$\varepsilon \left(\omega \right)\;=\varepsilon_{1}\left(\omega \right)+\varepsilon_{2}\left(\omega \right)$$

**Figure 7:**
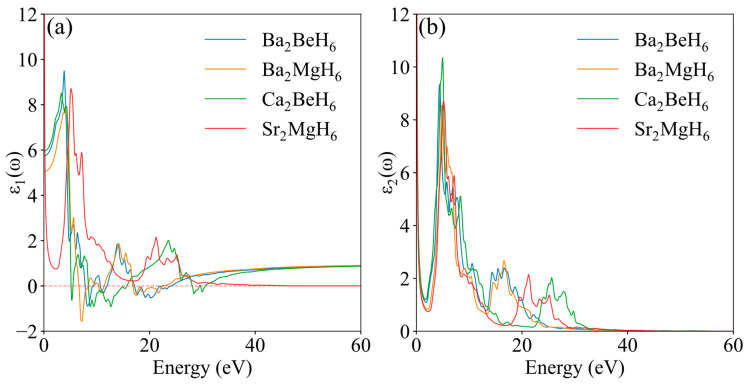
Calculated dielectric function of the X_2_MH_6_: (**a**) real part, and (**b**) imaginary part.

The real and imaginary components of the DF are represented by *ε*_1_(ω) and *ε*_2_(ω), respectively. The real part represents the extent of electrical polarization in a material under an external electric field, whereas the imaginary part reflects the material’s light absorption. Figure 7 shows the dielectric functions of the five structures as determined in this study.

Figure 7 illustrates the real part of the dielectric function, *ε*_1_(ω), reveals that all materials display significant positive peaks in the low-energy degree (0–10 eV), suggesting strong dielectric polarization responses to low-frequency light waves. The static value *ε*_1_(0) represents the material’s capability for a static dielectric response. From Figure 7, it is evident that Ca_2_BeH_6_ has the lowest static value, approximately 5.20, indicating its relatively weak static polarization ability. In the high-energy region (energies above 30 eV), *ε*_1_(ω) for Sr_2_MgH_6_ approaches 0, demonstrating that its polarization response to high-frequency light waves nearly vanishes. In comparison, the *ε*_1_(ω) values for the other four materials converge to approximately 0.95 at high frequencies, indicating their ability to maintain some polarization response in this region. All five materials show a primary peak at low frequencies and a secondary peak at high frequencies in the imaginary part of the dielectric function. The primary peaks are located at 5–15 eV, with Ca_2_BeH_6_ exhibiting the highest peak, indicating the strongest absorption. Regarding the secondary peaks, Ca_2_BeH_6_ shows the strongest absorption at 25.63 eV, whereas Sr_2_MgH_6_ peaks at 21.26 eV. The secondary peaks for the remaining three materials fall in the range of 15–18 eV.

**Figure 8:**
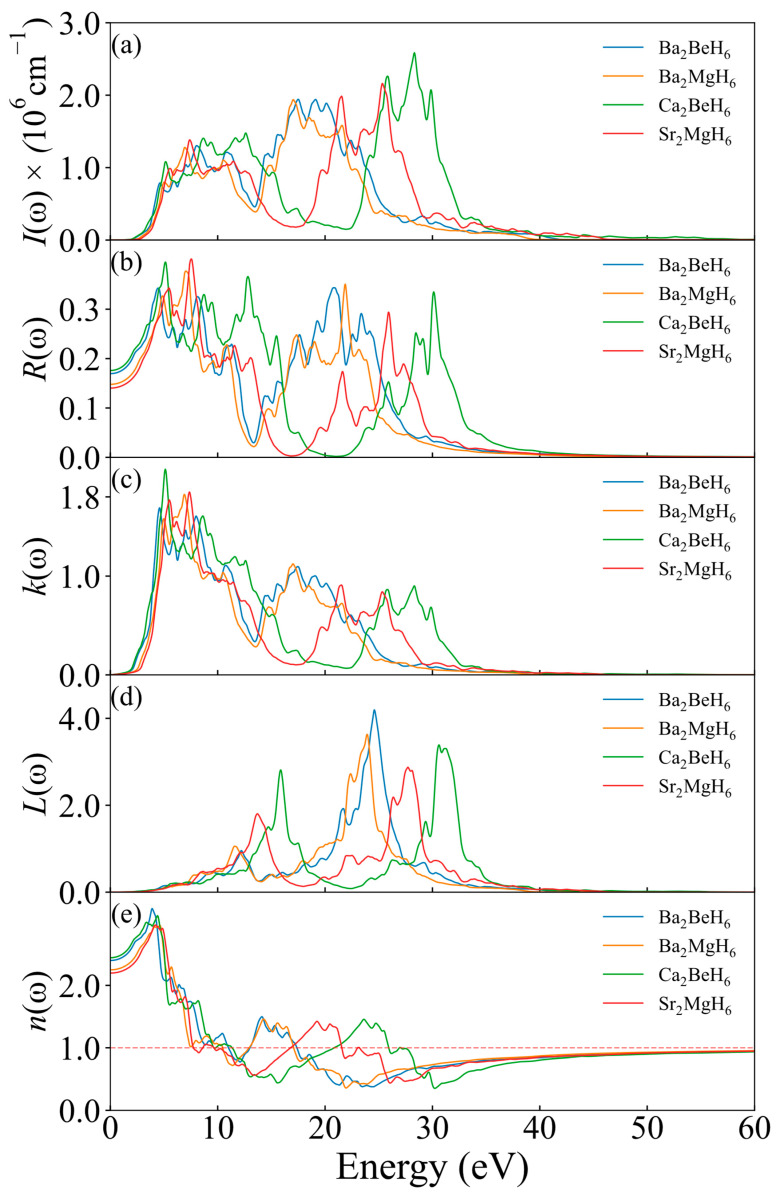
Calculated optical property of X_2_MH_6_ (**a**) absorption coefficient *I*(*ω*), (**b**) reflectivity *R*(*ω*), (**c**) extinction coefficient *K*(*ω*), (**d**) energy-loss spectrum *L*(*ω*), and (**e**) refractive index *n*(*ω*) of the X_2_MH_6_.

After discussing the materials’ DF, the optical properties of these five structures are shown in Figure 8. The following formulas are used to describe the absorption coefficient *I*(*ω*), reflectivity *R*(*ω*), extinction coefficient *K*(*ω*), energy-loss spectrum *L*(*ω*), and refractive index *n*(*ω*) [42,44]:

(14) $$I\;\left(\omega \right)\;={\;[\sqrt{{\left(\varepsilon_{1}\left(\omega \right)\right)}^{2}+{\left(\varepsilon_{2}\left(\omega \right)\right)}^{2}}+\varepsilon_{1}\left(\omega \right)]}^{\frac{1}{2}}$$

(15) $$R\;\left(\omega \right)=\frac{{\left(1-n^{2}\right)}^{2}+k^{2}}{{\left(1+n^{2}\right)}^{2}+k^{2}}$$

(16) $$K\;\left(\omega \right)=\frac{1}{\sqrt{2}}{\left[\sqrt{{\left(\varepsilon_{1}\left(\omega \right)\right)}^{2}+{\left(\varepsilon_{2}\left(\omega \right)\right)}^{2}}-\varepsilon_{1}\left(\omega \right)\right]}^{\frac{1}{2}}$$

(17) $$L\;\left(\omega \right)=\frac{\varepsilon_{2}\left(\omega \right)}{{\left(\varepsilon_{1}\left(\omega \right)\right)}^{2}+{\left(\varepsilon_{2}\left(\omega \right)\right)}^{2}}$$

(18) $$n\;\left(\omega \right)=\sqrt{\frac{\varepsilon_{1}\left(\omega \right)}{2}+\frac{\sqrt{{\left(\varepsilon_{1}\left(\omega \right)\right)}^{2}+{\left(\varepsilon_{2}\left(\omega \right)\right)}^{2}}}{2}}$$

The absorption and reflectivity of these materials were examined owing to their importance in solar energy applications. The curves of the absorption coefficient for the five structures closely mirror the trend of the Figure 7b, displaying two distinct peaks in both the low and high-frequency ranges. In the low-frequency range (5–15 eV), all materials show peaks. In the high-frequency range, Ca_2_BeH_6_ exhibits a peak at 25–30 eV, Sr_2_MgH_6_ shows a peak at 20 × 25 eV, while the remaining structures have their peaks in the 15–20 eV range. Reflectivity analysis indicates that all materials demonstrate relatively high reflectivity in the 0–10 eV range, with values around 34%. Ca_2_BeH_6_ displays peaks at 12.8 eV (37%) and 30.1 eV (33%). For the other structures, reflectivity peaks are noted in the 20–25 eV range, with Ba_2_MgH_6_ showing the highest reflectivity at 34%.

We compared the optical properties of the studied ordered-vacancy double perovskite structures with those of MAPbI_3_, a widely investigated archetypal organic–inorganic hybrid perovskite [45,46,47]. MAPbI_3_ exhibits a direct band gap of 1.5–1.6 eV, with strong optical response in the visible region (1.2–5.5 eV), and demonstrates excellent light absorption efficiency—about 80% of incident light can be absorbed with only ~280 nm film thickness—making it an ideal material for high-efficiency solar cells. In contrast, the ordered vacancy double perovskites possess wider band gaps (2.14–3.32 eV), with optical activity mainly in the ultraviolet region. These materials show characteristic dual-peak absorption and high reflectivity in the 5–30 eV range, with peak reflectance reaching up to 34%. Although the band gap of metal hydrides can be flexibly tuned via elemental substitution (with a variation up to 1.18 eV), their relatively large band gaps limit their potential for visible-light applications, rendering them more suitable for ultraviolet optoelectronic devices or protective coatings.

The extinction coefficient represents the material’s absorption characteristics and is usually related to the absorption and scattering processes of the material. From the graph, it can be seen that all the curves exhibit multiple peaks in different energy ranges, indicating that these materials have strong absorption at these specific energies. This is usually due to electronic transitions or vibrational modes in the materials. Overall, the extinction coefficient of all materials decreases with increasing energy, but in the high-energy region above 15 eV, all materials show one or two peaks. Among all the materials, Ca_2_BeH_6_ reaches peak values at 5.12 eV and 28.32 eV, with corresponding extinction coefficients of 2.08 and 0.90, making it the material with the highest peak in the low-energy region, while other materials’ peaks in the high-energy region occur at energies lower than those of Ca_2_BeH_6_.

From the energy loss spectrum *L*(*ω*), it can be observed that all materials exhibit distinct energy loss peaks in both the low-energy and high-energy regions, with the peaks in the high-energy region being higher than those in the low-energy region. This indicates that these materials experience strong light-matter interactions in different energy regions, leading to energy loss. Ca_2_BeH_6_ shows the strongest energy loss in the low-energy region (15 eV) and also has a noticeable peak in the high-energy region (32 eV), displaying relatively unique loss characteristics. On the other hand, Ba_2_BeH_6_ has the lowest peak in the low-energy region but the highest peak in the high-energy region among all the materials, suggesting that Ba_2_BeH_6_ exhibits significant light absorption and energy loss in the high-energy region, which may be related to its larger electronic transition amplitude or higher bandgap.

The refractive index describes the ratio of the speed of light in a material to the speed of light in a vacuum and is typically used to characterize the optical properties of materials. It is closely related to the interaction of the material with electromagnetic waves, as well as the direction and speed of light propagation. From the graph, it can be seen that the refractive index of all materials steadily increases within the 0–5 eV range. Around 5 eV, the refractive index of all materials reaches its peak, indicating strong interaction with light and slower light propagation. Between 5–10 eV, there is an overall downward trend, and at 10 eV, the refractive index for all materials is around 1. Then, in the 10–30 eV range, all materials show a small peak, with the peaks for Ca_2_BeH_6_ and Sr_2_MgH_6_ shifted to the “right” compared to the other two materials, with Ca_2_BeH_6_ having its second peak furthest to the right. When the energy exceeds 30 eV, the refractive index approaches 1 and becomes relatively stable, indicating that the optical properties of the materials in this region are relatively stable.

In summary, the optical and electronic properties of all X_2_MH_6_ materials indicate semiconductor characteristics with band gaps ranging from 2.14 to 3.32 eV, suitable for certain optoelectronic applications. They exhibit direct band gaps at the Γ point, facilitating efficient electronic transitions. Detailed analyses of the dielectric function, absorption coefficient, reflectivity, extinction coefficient, energy-loss spectrum, and refractive index reveal strong light–matter interactions in these materials. Among them, Ca_2_BeH_6_ shows the strongest optical response, including the highest absorption peaks and energy loss features, highlighting its superior optical performance. Compared with conventional organic–inorganic hybrid perovskites such as MAPbI_3_, these ordered-vacancy double perovskites have wider band gaps and optical activity shifted toward higher energies, making them less suitable for visible-light photovoltaic applications but promising for other optoelectronic devices and protective coatings.

## 4. Conclusions

To develop high-performance hydrogen storage materials, the properties of ordered vacancy double perovskite structures X_2_MH_6_ (Ba_2_BeH_6_, Ba_2_MgH_6_, Ca_2_BeH_6_, and Sr_2_MgH_6_) were systematically predicted using first-principles calculations. The stability of these materials was confirmed through formation energy calculations, Born stability criteria, and phonon spectra analysis. Hydrogen storage performance was evaluated by calculating gravimetric ($C_{wt}\%$) and volumetric ($\rho_{vol}$) hydrogen storage capacities. Among the five structures, Ca_2_BeH_6_ demonstrated the highest values at 6.32% for gravimetric capacity and 32.29 g∙H_2_/L for volumetric capacity, underscoring its exceptional hydrogen storage potential. It should be emphasized that the hydrogen storage capacities reported here are theoretical estimates based on structural models, and this work does not assess the practical storage and delivery performance of these materials. To assess their practical applicability, the key mechanical properties calculated include the bulk modulus *B*, Young’s modulus *E*, shear modulus *G*, Poisson’s ratio *v*, and the elastic anisotropy index *A*. Among the five structures, Ca_2_BeH_6_ exhibits the highest rigidity. These properties suggest that Ca_2_BeH_6_ is particularly well-suited for hydrogen storage applications. Additionally, the elastic anisotropy index *A* reveals weak anisotropy across all five materials. Their *B/G* ratios, ranging from 1.1 to 1.4, further confirm their favorable ductility. The Vickers hardness and melting point of the materials were also predicted to provide additional insights into their mechanical robustness. Band structure calculations reveal that these structures exhibit semiconducting properties, with band gaps of 3.11 eV (Ba_2_BeH_6_), 3.32 eV (Ba_2_MgH_6_), 2.14 eV (Ca_2_BeH_6_), and 2.87 eV (Sr_2_MgH_6_). Notably, all the four stable materials exhibited direct band gaps. The DOS analysis revealed that the s-orbitals of hydrogen atoms primarily influence the valence band, while the conduction band is mainly contributed by X atoms, with smaller but substantial contributions from M atoms, slightly surpassing those of hydrogen atoms. The optical properties of these materials were investigated by calculating their dielectric functions, absorption coefficients, refractive indices, extinction coefficients, energy-loss spectra, and reflectivity, revealing strong light–matter interactions. Among them, Ca_2_BeH_6_ exhibits the most pronounced optical response, including the highest absorption and energy-loss peaks. Compared to traditional hybrid perovskites such as MAPbI_3_, these ordered-vacancy double perovskites possess wider band gaps and blue-shifted optical activity, making them less suitable for visible-light photovoltaics but promising candidates for other optoelectronic applications and protective coatings. The results show that Ca_2_BeH_6_ exhibits exceptional physical properties due to its lowest formation energy, highest gravimetric hydrogen storage capacity, highest volumetric storage capacity, as well as a low Poisson’s ratio and high hardness, making it a promising candidate for hydrogen storage applications.

## Figures and Tables

**Table 1:** Optimized lattice constant, volume, formation energy, and elastic constants of X_2_MH_6_.

	Ba_2_BeH_6_	Ba_2_CaH_6_	Ba_2_MgH_6_	Ca_2_BeH_6_	Sr_2_MgH_6_
*a* (Å)	7.577	8.102	7.915	6.757	7.511
V (${Å}^{3}$)	435.023	531.921	495.765	308.527	423.737
${\Delta H}_{f}$ (eV/atom)	−0.75	−0.57	−0.74	−0.84	−0.75
*C*_11_ (GPa)	112	79	92	154	109
*C*_12_ (GPa)	26	17	20	25	20
*C*_44_ (GPa)	37	24	32	61	40

**Table 2:** Calculated hydrogen storage capacities of X_2_MH_6_.

	Ba_2_BeH_6_	Ba_2_MgH_6_	Ca_2_BeH_6_	Sr_2_MgH_6_
$C_{wt}\%$	1.47	1.98	6.32	2.91
$\rho_{vol}$	22.90	20.10	32.29	23.51

**Table 3:** Calculated elastic anisotropy *A*, bulk modulus *B* (GPa), shear modulus *G* (GPa), Young’s modulus *E* (GPa), *B/G* ratio, Poisson’s ratio ν, Vickers hardness (GPa) and melting point (*T_m_*) of the X_2_MH_6_ XAlH_3_ and XScH_3_ structures.

Structure	*A*	*B*	*G*	*E*	*B/G*	ν	*H*_Chen_	*T_m_*
Ba_2_BeH_6_	0.86	54.3	39.4	95.3	1.4	0.208	8.7	706.67 ± 300
Ba_2_MgH_6_	0.89	44.0	33.9	80.8	1.3	0.194	8.5	671.22 ± 300
Ca_2_BeH_6_	0.95	67.9	62.3	143.1	1.1	0.149	17.1	700.78 ± 300
Sr_2_MgH_6_	0.90	49.5	41.5	97.3	1.2	0.173	11.3	671.22 ± 300
KAlH_3_ [35]	0.14	1.19	11.61	8.79	0.10	−0.26		
RbAlH_3_ [35]	0.53	7.05	5.83	14.21	1.21	0.28		
CsAlH_3_ [35]	0.18	1.69	0.61	2.00	2.79	0.39		
KScH_3_ [36]	1.01	40.41	29.26	70.72	1.38	0.21		
RbScH_3_ [36]	1.13	39.04	29.41	70.52	1.33	0.20		
CsScH_3_ [36]	1.28	35.46	26.54	63.73	1.34	0.20		

## Data Availability

The raw data supporting the conclusions of this article will be made available by the authors upon request.

## References

[1] Khan M.I., Al-Ghamdi S.G. (2023). Hydrogen economy for sustainable development in GCC countries: A SWOT analysis considering current situation, challenges, and prospects. Int. J. Hydrogen Energy.

[2] Yang L., Cao Y., Xu Z., Qu N. (2025). First-principles prediction of hydrogen storage capabilities in Pd-based alkali metal hydride X_2_PdH_4_ (X = Na, K, Rb, and Cs). Int. J. Hydrogen Energy.

[3] Ahmed B., Tahir M.B., Ali A., Sagir M. (2024). DFT insights on structural, electronic, optical and mechanical properties of double perovskites X_2_FeH_6_ (X = Ca and Sr) for hydrogen-storage applications. Int. J. Hydrogen Energy.

[4] Ahmed B., Tahir M.B., Nazir S., Alzaid M., Ali A., Sagir M., Alrobei H. (2023). An Ab-initio simulation of boron-based hydride perovskites XBH_3_ (X = Cs and Rb) for advance hydrogen storage system. Comput. Theor. Chem..

[5] Al S. (2019). Theoretical investigations of elastic and thermodynamic properties of LiXH_4_ compounds for hydrogen storage. Int. J. Hydrogen Energy.

[6] Jena P. (2011). Materials for hydrogen storage: Past, present, and future. J. Phys. Chem. Lett..

[7] Lai Q., Paskevicius M., Sheppard D.A., Buckley C.E., Thornton A.W., Hill M.R., Gu Q., Mao J., Huang Z., Liu H.K. (2015). Hydrogen storage materials for mobile and stationary applications: Current state of the art. ChemSusChem..

[8] Hirscher M., Yartys V.A., Baricco M., Von Colbe J.B., Blanchard D., Bowman R.C., Broom D.P., Buckley C.E., Chang F., Chen P. (2020). Materials for hydrogen-based energy storage–past, recent progress and future outlook. J. Alloys Compd..

[9] Koufi A., Ziat Y., Belkhanchi H. (2024). Study of the Gravimetric, Electronic and Thermoelectric Properties of XAlH_3_ (X = Be, Na, K) as hydrogen storage perovskite using DFT and the BoltzTrap Software Package. Sol. Energy Sustain. Dev..

[10] Bouhadda Y., Rabehi A., Boudouma Y., Fenineche N., Drablia S., Meradji H. (2009). Hydrogen solid storage: First-principles study of ZrNiH_3_. Int. J. Hydrogen Energy.

[11] Chattaraj D., Dash S., Majumder C. (2016). Structural, electronic, elastic, vibrational and thermodynamic properties of ZrNi and ZrNiH_3_: A comprehensive study through first principles approach. Int. J. Hydrogen Energy.

[12] Simonovic B., Mentus S., Dimitrijevic R., Šusic M. (1999). Multiple hydriding/dehydriding of Zr_1.02_Ni_0.98_ alloy. Int. J. Hydrogen Energy.

[13] Korst W.L. (1962). The crystal structure of NiZrH_3_. J. Phys. Chem..

[14] Westlake D. (1980). Stoichiometries and interstitial site occupation in the hydrides of ZrNi and other isostructural intermetallic compounds. J. Less-Common. Met..

[15] Westlake D., Shaked H., Mason P., McCart B., Mueller M., Matsumoto T., Amano M. (1982). Interstitial site occupation in ZrNiH. J. Less-Common. Met..

[16] Zelai T. (2024). Study of magnetic, thermoelectric, and mechanical properties of double perovskites Be_2_XH_6_ (X = Cr and Mn) for spintronic and hydrogen-storage applications. Inorg. Chem. Commun..

[17] Guan S., Zhou J., Sun S., Peng Q., Guo X., Liu B., Zhou X., Tang Y. (2024). Nonmetallic Se/N Co-doped amorphous carbon anode collaborates to realize ultra-high capacity and fast potassium storage for potassium dual-ion batteries. Adv. Funct. Mater..

[18] Zhao X.H., Wei X.N., Tang T.Y., Gao L.K., Xie Q., Lu L.M., Tang Y.L. (2021). First-principles study on the structural, electronic and optical properties of vacancy-ordered double perovskites Cs_2_PtI_6_ and Rb_2_PtI_6_. Opt. Mater..

[19] Mubashir M., Ali M., Bibi Z., Younis M., Muzamil M. (2024). Efficient hydrogen storage in LiMgF_3_: A first principle study. Int. J. Hydrogen Energy.

[20] Kresse G., Furthmüller J. (1996). Efficient iterative schemes for ab initio total-energy calculations using a plane-wave basis set. Phys. Rev. B.

[21] Kresse G., Joubert D. (1999). From ultrasoft pseudopotentials to the projector augmented-wave method. Phys. Rev. B.

[22] Perdew J.P., Burke K., Ernzerhof M. (1996). Generalized gradient approximation made simple. Phys. Rev. Lett..

[23] Monkhorst H.J., Pack J.D. (1976). Special points for Brillouin-zone integrations. Phys. Rev. B.

[24] Cui Z., Sun Y., Li J., Qu J. (2007). Combination method for the calculation of elastic constants. Phys. Rev. B.

[25] Togo A., Tanaka I. (2015). First principles phonon calculations in materials science. Scr. Mater..

[26] Wang X., Wei Q., Luo J., Jia X., Zhang M., Zhu X., Wei B. (2025). Pressure-Induced Phase Transitions and Electronic Structure Evolution of Ba_4_Au. Materials.

[27] Saal J.E., Kirklin S., Aykol M., Meredig B., Wolverton C. (2013). Materials design and discovery with high-throughput density functional theory: The open quantum materials database (OQMD). JOM.

[28] Kirklin S., Saal J.E., Meredig B., Thompson A., Doak J.W., Aykol M., Rühl S., Wolverton C. (2015). The Open Quantum Materials Database (OQMD): Assessing the accuracy of DFT formation energies. npj Comput. Mater..

[29] Mustafa G.M., Younas B., Alkhaldi H.D., Mera A., Alqorashi A.K., Hakami J., Mahmoud S.A., Boukhris I., Mahmood Q. (2024). First principle study of physical aspects and hydrogen storage capacity of magnesium-based double perovskite hydrides Mg_2_XH_6_ (X = Cr, Mn). Int. J. Hydrogen Energy.

[30] Baysal M., Surucu G., Deligoz E., Ozısık H. (2018). The effect of hydrogen on the electronic, mechanical and phonon properties of LaMgNi_4_ and its hydrides for hydrogen storage applications. Int. J. Hydrogen Energy.

[31] Gencer A., Surucu G. (2019). Investigation of structural, electronic and lattice dynamical properties of XNiH_3_ (X = Li, Na and K) perovskite type hydrides and their hydrogen storage applications. Int. J. Hydrogen Energy.

[32] Gencer A., Surucu G. (2020). Enhancement of hydrogen storage properties of Ca_3_CH antiperovskite compound with hydrogen doping. Int. J. Energy Res..

[33] Baaddi M., Chami R., Baalla O., Quaoubi S.E., Saadi A., Omari L.E.H., Chafi M. (2024). The effect of strain on hydrogen storage characteristics in K_2_NaAlH_6_ double perovskite hydride through first principle method. Environ. Sci. Pollut. Res..

[34] Pugh S.F. (1954). XCII. Relations between the elastic moduli and the plastic properties of polycrystalline pure metals. Lond. Edinb. Dublin Philos. Mag. J. Sci..

[35] Umer M., Murtaza G., Ahmad N., Ayyaz A., Raza H.H., Usman A., Liaqat A., Manoharadas S. (2024). First principles investigation of structural, mechanical, thermodynamic, and electronic properties of Al-based perovskites XAlH_3_ (X=K, Rb, Cs) for hydrogen storage. Int. J. Hydrogen Energy.

[36] Xu N., Song R., Chen S., Chen Y., Li S., Jiang Z. (2025). First-principles study on the structure, mechanical, electrical, optical, kinetic, thermodynamic and hydrogen storage properties of the hydride perovskites XScH_3_ (X = K, Rb, Cs) for hydrogen storage applications. J. Energy Storage.

[37] Luo J., Zhang M., Jia X., Wei Q. (2025). Study of the stable structures and properties of Ru_2_Al_5_. Chin. Phys. B.

[38] Wei Q., Yang J., Jia X., Luo J., Zhang M., Zhu X. (2025). Crystal structures, mechanical properties, and electronic structure analysis of ternary FeCrAl alloys. Phys. Lett. A.

[39] Chen X.Q., Niu H., Li D., Li Y. (2011). Modeling hardness of polycrystalline materials and bulk metallic glasses. Intermetallics.

[40] Yan H., Zhang W., Chen L., Zhang Y., Wang H., Zhang M., Wei Q. (2025). Structural, strength and fracture mechanisms of superconducting transition metal nitrides TM_3_N_5_ (TM = W and Mo). Phys. Chem. Chem. Phys..

[41] Fine M., Brown L., Marcus H. (1984). Elastic constants versus melting temperature in metals. Scr. Metall..

[42] Hussain S., Rehman J.U., Tahir M.B., Hussain A. (2024). First-principles study of structural, mechanical, optical, and electronic properties of double perovskite RbBa_2_Ti_3_O_10_ material for photocatalytic applications. Int. J. Hydrogen Energy.

[43] Blöchl P.E. (1994). Projector augmented-wave method. Phys. Rev. B.

[44] Gupta S.L., Kumar S., Panwar S. (2024). Ab initio studies of newly proposed zirconium based novel combinations of hydride perovskites ZrXH_3_ (X = Zn, Cd) as hydrogen storage applications. Int. J. Hydrogen Energy.

[45] Shockley W., Queisser H.J. (1961). Detailed Balance Limit of Efficiency of *p-n* Junction Solar Cells. J. Appl. Phys..

[46] Roldan-Carmona C., Malinkiewicz O., Betancur R., Longo G., Momblona C., Jaramillo F., Camacho L., Bolink H.J. (2014). High efficiency single-junction semitransparent perovskite solar cells. Energy Environ. Sci..

[47] Leguy A.M.A., Azarhoosh P., Alonso M.I., Campoy-Quiles M., Weber O.J., Yao J., Bryant D., Weller M.T., Nelson J., Walsh A. (2016). Experimental and theoretical optical properties of methylammonium lead halide perovskites. Nanoscale.

